# Investigation of Community Behaviors, Socioeconomic Factors, and Breakthrough COVID-19 Infections Among Vaccinated Individuals: Cross-Sectional Study

**DOI:** 10.2196/76679

**Published:** 2026-04-01

**Authors:** Matthew J McDonald, Bathri Narayan Vajravelu

**Affiliations:** 1 Department of Physician Assistant Studies, Massachusetts College of Pharmacy and Health Sciences, Boston, MA, United States, 1 617-732-2961

**Keywords:** high-risk behaviors, vaccinated individuals, socioeconomic factors, breakthrough infections, COVID-19, social distancing

## Abstract

**Background:**

Despite widespread COVID-19 vaccination, breakthrough infections remain a public health concern, with transmission risks potentially linked to community behaviors and age-specific preventive practices. While mask-wearing and social distancing are well-established mitigation strategies, their adoption patterns across age groups, particularly among vaccinated individuals, are poorly understood.

**Objective:**

This study focuses on understanding breakthrough infections among vaccinated individuals, high-risk behaviors, and socioeconomic determinants of COVID-19 susceptibility to guide effective public health interventions.

**Methods:**

A 31-question voluntary survey was distributed using convenience sampling through the Qualtrics survey platform. All survey respondents reported receiving at least the primary vaccination against COVID-19 infection, and all survey responses were recorded between January 6, 2022, and September 26, 2022. Logistic regression analysis was used to estimate the odds ratio to measure the association between testing positive for COVID-19 and the different activities.

**Results:**

Among the vaccinated individuals, those who tested positive were 11.103 times more likely to engage in going to a restaurant or bar compared to those who tested negative (*P*=.01). There was a significant difference in practicing social distancing and mask-wearing between the different age groups (*P*=.02), with 100% (10/10) of the participants older than 70 years practicing it, followed by 96.8% (118/122) of the 18 to 29 year olds. The study found lower infection rates in the same age groups compared to the other age groups. Moreover, the 18 to 29 years age group demonstrated notable associations with practicing social distancing and mask-wearing in various settings.

**Conclusions:**

Compliance with social distancing and mask-wearing was higher among older and younger participants, and noncompliance with social distancing and mask-wearing was associated with a higher positivity rate. Activities such as going to a restaurant or bar were significantly associated with testing positive for COVID-19 among vaccinated individuals. These results provide valuable information to individuals, health care providers, and public health experts regarding the types of behaviors and community settings that are associated with COVID-19 infection and help enhance our understanding of the types of settings in which social distancing and masking may be beneficial or not necessary. This knowledge can also help local health departments develop tailored public health guidance based on the behaviors of individuals and the types of community settings in their localities.

## Introduction

Since late 2019, the emergence of coronavirus disease 2019, commonly known as COVID-19, has disrupted much of society and resulted in over 1.1 million reported deaths in the United States [1]. Early in the pandemic, the Centers for Disease Control and Prevention (CDC), local public health departments, and other public health authorities sought to mitigate transmission and potential loss through various public health measures, which included public masking requirements, social distancing, lockdowns, and school and business closures.

The transmission of COVID-19 is variable and dependent on many factors, including the type of contact with an individual who is infected, duration of exposure, host immune factors, and ventilation [2]. The dominant route of transmission of COVID-19 is respiratory, and it is known that COVID-19 spreads effectively in enclosed and crowded settings [3]. Masking and social distancing have been shown to reduce transmission of COVID-19 in many settings [4-6].

The Omicron variants, known for increased transmissibility and immune evasion from both natural and vaccine-induced immunity, were discovered in 2022 and became the dominant variant across the world in 2023 [7]. The CDC tracked new cases of COVID-19 until early 2023. Guidance on personal protective measures had previously been based on community levels of circulating COVID-19 infections. However, in 2023, the CDC changed its data collection and reporting, focusing on the weekly number of hospitalizations and deaths rather than new cases of infection, as well as monitoring variants based on data from a limited number of areas in the United States [8]. With the dominance of Omicron, COVID-19 vaccine formulations were updated from previously protecting against the XBB sublineage strains to providing protection against the Omicron JN.1 lineage [9]. On June 27, 2024, the CDC’s Advisory Committee on Immunization Practices recommended vaccination with a Food and Drug Administration–approved or authorized vaccine for all persons aged 6 months or older [9]. Current Food and Drug Administration–approved vaccinations for COVID-19 prevention include vaccines by Moderna, Pfizer-BioNTech, and Novavax. Prior COVID-19 infection as well as COVID-19 vaccination have been shown to reduce the severity and transmission of COVID-19 infection, and this may impact the accuracy of CDC COVID-19 data collection [10].

Many of the stringent public health restrictions implemented early in the pandemic have been replaced by less strict, practical measures that place the responsibility of social distancing and masking when sick on individuals in conjunction with local transmission rates [2]. In March 2024, the CDC released updated recommendations for individuals to prevent the spread of upper respiratory viruses from common respiratory viruses, including COVID-19, influenza, and respiratory syncytial virus, intending to simplify public health recommendations rather than have separate pathogen-specific guidelines [9]. The guidelines allow individuals who have confirmed or suspected COVID-19 infection to resume normal activities if their symptoms are improving and they have been afebrile (without antipyretics) for at least 24 hours. They are encouraged to take additional prevention strategies for the next 5 days, such as masking, enhancing hygiene practices, and social distancing [9]. With newer guidelines allowing individuals the ability to re-enter the community while still symptomatic and potentially infectious with upper respiratory viruses, including COVID-19, research pertaining to specific community settings and behaviors that increase one’s risk for infection is necessary to help individuals reduce their own risk of infection [11].

With the availability of COVID-19 vaccines, understanding the types of community behaviors and settings that are associated with a high risk of COVID-19 transmission in vaccinated individuals is an important area of research because individuals can make more informed decisions about the types of behaviors and settings they are willing to engage in based on the risk of infection even after vaccination. Individuals will be better informed about the potential risk of infection, allowing them to determine whether personal protective measures to reduce the risk of infection, such as social distancing or masking, are more appropriate in specific settings. For policymakers and public health experts, this study will provide information about the behaviors and settings that are associated with a risk of COVID-19 infection, allowing them to develop tailored infection prevention strategies and guidance for community members pertaining to COVID-19 and other respiratory viral infections.

This study aims to identify community behaviors, settings, and close contact exposures of vaccinated individuals that are associated with testing positive for COVID-19 despite vaccination status. Additionally, this study sought to evaluate any socioeconomic factors associated with COVID-19 infection, including demographics, household income, and type of residence. The authors’ hypothesis is that certain community behaviors that have the potential to be crowded and have poor ventilation, such as indoor restaurants, bars, cafés, places of worship, fitness centers, entertainment venues, and using public transportation, will be associated with a higher breakthrough risk of COVID-19 infection.

## Methods

### Ethical Considerations

The institutional review board of Massachusetts College of Pharmacy and Health Sciences (MCPHS) in Boston, MA, reviewed and approved this cross-sectional research study on November 19, 2021 (IRB-2021-2022-39). This study was conducted in accordance with the Belmont Report ethical principles of respect for persons, beneficence, and justice, and in compliance with the Federal Policy for the Protection of Human Subjects (45 CFR 46, The Common Rule). The study also adhered to the principles of the Declaration of Helsinki and the STROBE (Strengthening the Reporting of Observational Studies in Epidemiology) guidelines (Multimedia appendices 1). Informed consent was obtained electronically from all participants prior to their participation via the Qualtrics online survey platform. Participants were informed of the study's purpose, voluntary nature, and their right to withdraw at any time without consequence. No compensation was provided for participation. To protect participant privacy and confidentiality, no personally identifiable information was collected at any point during the study. All survey responses were collected anonymously through Qualtrics, which was configured to disable the collection of IP addresses and other identifying metadata. Data were stored securely on password-protected institutional servers, accessible only to the research team. All data were analyzed in aggregate and reported in a manner that prevents the identification of individual participants.

### Study Design and Data Collection

An online survey was created using Qualtrics, and all survey responses were recorded between January 6, 2022, and September 26, 2022. The survey items related to sociodemographic characteristics, community behaviors, and personal preventive measures were adapted from previously validated survey instruments [12-14]. The survey was pilot-tested among a small group of faculty members and students at MCPHS to confirm the clarity and validity of the tool before distribution. The reliability of the survey was assessed using the Cronbach α coefficient, which was calculated to be 0.87, indicating good internal consistency among the survey items. Convenience sampling was used to recruit respondents for the survey and included email, social media postings, and in-person recruitment. No formal sample size calculation or power analysis was performed before the study since this was an exploratory cross-sectional study using convenience sampling. The final sample size achieved was adequate for the regression analysis for the number of outcome events and the predictor variables included in the model. Approximately 823 individuals were recruited by email, and 5 social media postings were disseminated using Blackboard and Facebook. The recruitment emails and social media postings included information about the study and the survey link to participate. The social media postings targeted students and faculty at MCPHS. Recruitment emails targeted department heads and faculty members from several United States medical universities and physician assistant programs. The publicly available email addresses of department heads and faculty members from several United States medical universities and physician assistant programs were gathered from their respective university websites. Survey links could then be disseminated to other eligible individuals at their discretion. The survey consisted of a range of 22 to 31 questions. Survey items on frequency of activities (eg, visiting restaurants, coffee shops, gyms, and places of worship) used a 5-point Likert scale (Never, Rarely, Sometimes, Often, Always). For regression analyses, these responses were dichotomized into “Never/Rarely/Sometimes” versus “Most of the time/Always.” This approach was chosen to improve model stability and ensure sufficient sample size within each category. Inclusion criteria included being at least 18 years old, and each respondent reported receiving at least the primary vaccination against COVID-19 infection. Primary vaccination included the original vaccine regimens in the United States: 2 doses of the original mRNA vaccines by Pfizer-BioNTech or Moderna, or 1 dose of the Johnson & Johnson vaccine. Respondents were included if they reported receiving a vaccine regimen approved in a non-US country, but the type of vaccine was not collected. A survey answer option of “Other” was included in the question where respondents were asked about receiving a vaccine that was not specifically listed. COVID-19 positivity was self-reported and based on a positive COVID-19 PCR or rapid antigen test anytime during the study period. The survey was closed, and no further responses were recorded once a second booster shot was authorized, as our survey did not include questions regarding a second booster, which could confound the study’s results.

### Statistical Analysis

Logistic regression analysis was used to estimate the odds of COVID-19 infection in relation to various risk factors identified within the survey dataset. The primary outcome variable was self-reported COVID-19 positivity (yes/no), and the primary exposures were self-reported frequency of participation in community activities (eg, shopping, dining at restaurants, bars, attending gyms, salons, air travel, public transportation, indoor entertainment, places of worship, and social gatherings), masking, and social distancing practices. Predictor variables were sociodemographic variables such as age, sex, and occupational exposure. Potential confounders were age and occupational exposure. There were no identified effect modifiers. Odds ratios with 95% CIs were reported for each analysis. Both univariable and multivariable logistic regression models were conducted. In the multivariable analyses, sociodemographic factors and behavioral risk variables were entered simultaneously to assess their independent associations with COVID-19 positivity. To minimize overfitting, we performed separate multivariable models focusing on conceptually grouped predictors (eg, community activities, social distancing behaviors, sociodemographic variables). Sociodemographic factors were not included in the same model as activity variables because of collinearity concerns and sample size limitations. This analytic strategy allowed a clearer interpretation of specific domains of risk. Respondents who did not complete the entire survey were removed from the data analysis. A *P* value of less than .05 was established for statistical significance. IBM SPSS Statistics version 29.0 (IBM Corp) was used for statistical analysis.

## Results

The primary goal of this investigation is to unveil the community behaviors, settings, and close contact scenarios correlated with positive COVID-19 test results among vaccinated individuals. Additionally, the study explores the socioeconomic factors related to COVID-19 infection, including demographics, household income, and type of residence. A total of 297 participants completed the survey, of whom 25 were excluded because of missing data. Among the remaining 272 participants who answered the entire survey, 208 (76.5%) were female, 62 (22.8%) were male, and 2 (0.8%) identified as either nonbinary, third gender, or preferred not to say. The precise response rate is unclear because the researchers were unable to determine how many participants received the survey as a result of email or social media recruitment. The majority of respondents to the survey fell within the 18 to 29 years age group, constituting 44.9% (122/272) of participants, a demographic typically representative of college students. Furthermore, a substantial proportion of the survey participants identified as White, accounting for 83.8% (228/272), as shown in Table 1.

**Table 1.** Demographic characteristics of vaccinated adult participants in a cross-sectional online survey assessing breakthrough COVID-19 infections in the United States between January 2022 and September 2022.

Characteristic	Participants, n (%)
Gender	
Male	62 (22.8)
Female	208 (76.5)
Nonbinary/third gender	1 (0.4)
Prefer not to say	1 (0.4)
Age group (y)	
18‐29	122 (44.9)
30‐39	39 (14.3)
40‐49	39 (14.3)
50‐59	30 (11)
60‐69	31 (11.4)
>70	10 (3.7)
Race or ethnicity	
Asian Indian	12 (4.4)
Black or African American	7 (2.6)
Chinese	5 (1.8)
Filipino	1 (0.4)
Hispanic or Latino	9 (3.3)
Multiracial or biracial	3 (1.1)
Native American or Alaskan Native	2 (0.7)
Vietnamese	1 (0.4)
White	228 (83.8)
Other race not listed	2 (0.7)

A logistic regression was used to assess the impact of community activities on the likelihood of COVID-19 infection in vaccinated individuals. The model exhibited statistical significance (*χ*^2^_11_=25.048; *P*<.001), indicating its ability to elucidate 33% (Nagelkerke *R*^2^) of the variance in COVID-19 among vaccinated individuals and correctly classify 79.2% (215/272) of cases. The analysis provided insights into the influence of specific activities (see Table 2) on COVID-19 infection in vaccinated individuals. Notably, those who tested positive were 11.103 times more likely (*P*=.01) to engage in going to a restaurant or bar compared with those who tested negative (Table 2). These results suggest a significant association between community activities and COVID-19 infection in vaccinated individuals, emphasizing the importance of specific behaviors in influencing infection outcomes.

**Table 2.** Association between community activities and self-reported COVID-19 positivity among vaccinated adults who participated in the online cross-sectional survey in the United States between January 2022 and September 2022.

Activity	OR^a^ (95% CI)	*P* value
Shopping for items	0.99 (0.27‐3.65)	.99
Place of worship	0.24 (0.07‐0.91)	.04
Restaurant or bar	11.10 (1.80‐68.57)	.01
Coffee shop	0.77 (0.24‐2.44)	.66
Public transport	0.24 (0.06‐1.02)	.05
Airplane travel	0.49 (0.14‐1.73)	.27
Office setting	3.08 (0.60‐15.86)	.18
Gym or fitness	0.14 (0.02‐0.93)	.04
Salon or barber	1.56 (0.47‐5.17)	.47
Vehicle travel	2.39 (0.72‐7.98)	.16
Indoor entertainment	0.35 (0.10‐1.26)	.11

a OR: odds ratio.

Among the different age groups who responded to the survey, we found that infection rates were the lowest in the more than 70 years age group (30%; n=3) and the 18 to 29 years age group (40.7%; n=49) compared to the other age groups, although the difference did not achieve statistical significance. Then, we sought to determine whether there was any difference in the positivity rate associated with a specific activity between respondents who reported social distancing or wearing their masks most of the time and those who did not do so most of the time. The initial model, which included no predictors (null model), displayed a classification table with an overall correct classification rate of 59.9% (163/272), indicating suboptimal performance in predicting COVID-19 positivity based on the specified variables.

After incorporating predictors from the question that asked about “social distancing and mask-wearing behaviors in specific settings,” the model showed statistically insignificant results (*χ*^2^_11_=17.612; *P* >.05). The model elucidated only 8.5% (Nagelkerke *R*^2^) of the variance in COVID-19 positivity among vaccinated individuals and accurately classified 60.7% (165/272) of cases. A detailed examination of the results (Table 3) indicated that none of the predictors achieved statistical significance. For example, participants who tested positive were 3.001 times more likely to go to an office setting compared to those who did not test positive, but this result did not reach statistical significance (*P*=.09).

**Table 3.** Association between masking and social distancing and self-reported COVID-19 positivity among vaccinated adults who participated in the online cross-sectional survey in the United States between January and September 2022.

Activity	OR^a^ (95% CI)	*P* value
Shop for items	0.54 (0.19‐1.57)	.26
Place of worship	1.29 (0.42‐3.94)	.65
Restaurant or bar	1.71 (0.48‐6.11)	.41
Coffee shop	1.38 (0.39‐4.88)	.62
Public transport	1.71 (0.46‐6.29)	.42
Airplane travel	1.77 (0.52‐6.04)	.36
Office setting	3.00 (0.85‐10.65)	.09
Gym or fitness	1.09 (0.36‐3.30)	.88
Salon or barber	0.87 (0.27‐2.78)	.82
Vehicle travel	1.46 (0.45‐4.72)	.53
Indoor entertainment	0.25 (0.07‐0.87)	.03

a OR: odds ratio.

In summary, the logistic regression model did not provide substantial evidence of a significant difference in COVID-19 positivity rates based on specific activities among respondents reporting varying levels of social distancing and mask-wearing. The overall model did not achieve statistical significance, and the predictors included did not significantly contribute to explaining the variability in COVID-19 positivity.

We then tested whether any of the following demographic variables were associated with a higher COVID-19 positivity rate, including household income, type of residence, education level, race, and number of individuals living in a household. The logistic regression aimed to investigate whether specific demographic variables were correlated with a higher COVID-19 positivity rate among participants. The initial model, representing the null model with no predictors, exhibited a classification table with an overall correct classification rate of 59.9% (163/272), indicating suboptimal performance in predicting COVID-19 positivity based on the specified variables. Subsequent inclusion of demographic variables also yielded statistically insignificant findings (*χ*^2^_22_=20.573; *P*>.05). The model elucidated 10.6% (Nagelkerke *R*^2^) of the variance in COVID-19 positivity among participants and accurately classified 61% (166/272) of cases. The analysis of the demographic variables (Table 4) revealed that none of the predictors achieved statistical significance. This suggests that none of the demographic variables were independently associated with a higher COVID-19 positivity rate.

**Table 4.** Association between sociodemographic factors and self-reported COVID-19 positivity among vaccinated adults who participated in the online cross-sectional survey in the United States between January 2022 and September 2022.

Variable	OR^a^ (95% CI)	*P* value
Annual income (US $)		
Under 25,000	1.14 (0.42‐3.09)	.79
25,000‐49,999	0.43 (0.11‐1.68)	.22
50,000‐74,999	1.33 (0.41‐4.36)	.64
75,000‐99,999	0.66 (0.17‐2.64)	.56
100,000‐149,999	1.22 (0.35‐4.32)	.75
150,000‐199,999	1.09 (0.41‐2.92)	.86
Over $200,000	0.64 (0.19‐2.19)	.48
Residence type		
Single-family house	1.15 (0.21‐6.31)	.87
Two-family home or duplex	0.92 (0.49‐1.73)	.79
Apartment or Condo	0.00 (0.00-∝)	>.99
Education		
High school	2.28 (0.00-∝)	>.99
Some college—no degree	2,082,843,782.47 (0.00-∝)	>.99
Associate degree	1.18 (0.26‐5.40)	.84
Bachelor’s degree	1.81 (0.39‐8.39)	.45
Graduate degree	2.63 (0.21‐32.61)	.45
Ethnicity		
White	0.00 (0.00-∝)	>.99
Black/African American	0.31 (0.06‐1.63)	.17
Hispanic/Latino	1.37 (0.25‐7.39)	.71
Native American/Alaskan Native	1.03 (0.15‐7.16)	.98
Asian Indian	0.00 (0.00-∝)	>.99
Multiracial/Biracial	0.37 (0.07‐1.90)	.23
Chinese	0.00 (0.00-∝)	>.99
Filipino	1.12 (0.04‐28.39)	.95
Vietnamese	1.33 (0.08‐22.78)	.85
Other race not listed	0.00 (0.00-∝)	>.99

a OR: odds ratio.

In summary, the logistic regression model revealed no statistically significant associations between demographic variables (eg, income, education, and race) and COVID-19 positivity in our vaccinated cohort. While this suggests that socioeconomic factors alone may not independently predict breakthrough infections in this population, it underscores the potential dominance of behavioral or environmental determinants (eg, activity-related risks, as identified in our other analyses).

We then investigated whether living with someone who worked in health care was associated with a higher positivity rate. Contrary to expectations, our analysis found no significant association between living with a health care worker and COVID-19 positivity rates among vaccinated individuals (Table 5). This suggests that, in our cohort, household exposure to health care personnel, who likely had frequent occupational exposure to SARS-CoV-2, did not translate into a higher infection risk for vaccinated cohabitants. These findings may reflect the success of infection control measures in health care settings, the protective effect of vaccination in household contexts, or potential compensatory behaviors (eg, stricter hygiene practices) in these households. While reassuring, these results should be interpreted in context: the study period (2022) coincided with widespread vaccine availability and improved workplace protections, which may have mitigated risks that were more prominent earlier in the pandemic.

**Table 5.** Association between household exposure to health care workers and self-reported COVID-19 positivity among vaccinated adults who participated in the online cross-sectional survey in the United States between January 2022 and September 2022.

Household health care workers	OR^a^ (95% CI)	*P* value
None	0.00 (0.00-∝)	>.99
One	1.15 (0.65‐2.03)	.64
More than one	0.91 (0.38‐2.19)	.83

a OR: odds ratio.

We also tested whether there was an association between a specific vaccine (Moderna, Pfizer, or Johnson & Johnson) and a higher or lower hospitalization rate among respondents. The logistic regression model did not yield evidence of a significant association between the type of vaccine and the hospitalization rate among participants. The overall model was statistically insignificant, and the predictor included in the model did not substantially contribute to explaining the variability in hospitalization rates among the participants (Table 6). This indicates that all 3 vaccines are equally effective in reducing hospitalizations due to COVID-19.

**Table 6.** Association between type of COVID-19 vaccine and hospitalization rate among vaccinated adults who participated in the online cross-sectional survey in the United States between January 2022 and September 2022.

Vaccine	OR^a^ (95% CI)	*P* value
Moderna	0.98 (0.27‐3.61)	.98
Pfizer-BioNTech	2.21 (0.60‐8.12)	.23
Johnson & Johnson	0.87 (0.51‐1.47)	.60

a OR: odds ratio.

We then analyzed the relationship between distinct age groups and their adherence to social distancing and mask-wearing practices in various scenarios. We identified a statistically significant difference among the different age groups in practicing social distancing and mask-wearing practices (*P*=.02). The highest prevalence was found in the older than 70 years age group, where 100% of the respondents reported social distancing practices, followed by the 18 to 29 years age group (96.8%). The results reveal significant associations, particularly within the 18 to 29 age group, consistently exhibiting a higher likelihood of practicing these preventive measures across diverse settings. When it comes to shopping for items, individuals aged 18 to 29 consistently showed a significant association with “Always or Most of the time,” engaging in social distancing and mask-wearing. Similarly, this age group displayed significant associations in scenarios involving people gathering indoors, whether with more than 10 people or with 10 people or fewer.

Moreover, the 18 to 29 age group demonstrated notable associations with practicing social distancing and mask-wearing in various settings, including attending indoor church or religious gatherings, going to restaurants or bars, visiting coffee shops, using public transportation, traveling via airplane, going to an office setting (excluding health care purposes), going to a gym or fitness center, and visiting a salon or barber. The consistent pattern emphasizes the inclination of the 18 to 29 age group to adhere to social distancing and mask-wearing guidelines across a spectrum of activities. The statistical significance of these associations, as indicated by the *P* value, underscores the robustness of these observed patterns. These insights can inform targeted public health interventions and communication strategies, recognizing the variations in behavior across different age demographics (Table 7).

**Table 7.** Association between age groups and their adherence to social distancing and mask-wearing practices in various community settings among vaccinated adults who participated in the online cross-sectional survey in the United States between January 2022 and September 2022.

Activity and age group (y)		Never or rarely or sometimes	Always or most of the time	*P* value
Shop for items (groceries, prescriptions, home goods, and clothing)				<.001
>70		1	9	
18‐29		24	98	
30-39		2	37	
40‐49		0	39	
50‐59		0	30	
60‐69		1	30	
Have people visit you inside your home or go inside someone else’s home where there were more than 10 people				<.001
>70		2	8	
18‐29		43	79	
30-39		2	37	
40‐49		0	39	
50‐59		1	29	
60‐69		1	30	
Have people visit you inside your home or go inside someone else’s home where there were 10 people or less				<.001
>70		1	9	
18‐29		35	87	
30-39		2	37	
40‐49		1	38	
50‐59		2	28	
60‐69		2	29	
Go to an indoor church or a religious gathering or place of worship				<.001
>70		0	10	
18‐29		33	89	
30-39		2	37	
40‐49		0	39	
50‐59		2	28	
60‐69		2	29	
Go to a restaurant or bar (dine-in, any area designated by the restaurant including patio seating)				<.001
>70		2	8	
18‐29		29	93	
30-39		1	38	
40‐49		0	39	
50‐59		1	29	
60‐69		1	30	
Go to a coffee shop				<.001
>70		1	9	
18‐29		28	94	
30-39		1	38	
40‐49		0	39	
50‐59		0	30	
60‐69		1	30	
Use public transportation (bus, subway, streetcar, and train)				<.001
>70		1	9	
18‐29		26	96	
30-39		1	38	
40‐49		0	39	
50‐59		1	29	
60‐69		2	29	
Travel via airplane				<.001
>70		2	8	
18‐29		42	80	
30-39		2	37	
40‐49		0	39	
50‐59		2	28	
60‐69		1	30	
Go to an office setting (other than for health care purposes)				<.001
>70		0	10	
18‐29		36	86	
30-39		2	37	
40‐49		2	37	
50‐59		1	29	
60‐69		1	30	
Go to a gym or fitness center				<.001
>70		0	10	
18‐29		40	82	
30-39		1	38	
40‐49		1	38	
50‐59		2	28	
60‐69		2	29	
Go to a salon or barber (eg, hair salon and nail salon)				<.001
>70		1	9	
18‐29		43	79	
30-39		2	37	
40‐49		0	39	
50‐59		0	30	
60‐69		2	29	

## Discussion

### Overview

Determining the type of community activities and settings that are associated with COVID-19 in vaccinated individuals is important for individuals to assess the risk of the activities they are willing to engage in. Identifying the behaviors and community settings that are associated with COVID-19 infection will also help enhance our understanding of the types of settings in which social distancing and masking may be more efficacious. This knowledge can help local health departments develop tailored public health guidance based on the behaviors of individuals and the types of community settings in their localities.

### Principal Results

One of the primary goals of this study was to reveal community behaviors linked to positive COVID-19 test results among vaccinated individuals. The present study demonstrates that vaccinated individuals were more likely to get infected with the virus while visiting a restaurant or a bar, emphasizing the importance of specific behaviors in shaping infection outcomes. This explains the infection rate among vaccinated individuals and aligns with the results reported by Zhang et al [15,16], who found higher infection rates among people visiting a restaurant. This might be a result of inadequate ventilation, which could lead to a rise in the concentration of airborne SARS-CoV-2. The current research revealed that individuals from the youngest (18‐29 y) and oldest (>70 y) demographics were notably diligent about practicing social distancing behavior and mask-wearing, potentially linked to elevated levels of COVID-19-related anxiety among this cohort. This might also stem from the fact that individuals in the youngest age group are highly socially connected and actively engaged in extensive community interactions [14].

We sought to examine the difference in positivity rates related to specific activities between respondents who reported adhering to social distancing and wearing masks most of the time and those who did not. Nevertheless, our analysis did not provide strong evidence of a significant difference in COVID-19 positivity rates based on specific activities among respondents with varying levels of social distancing and mask-wearing. The overall model did not achieve statistical significance, and the predictors included did not substantially contribute to explaining the variability in COVID-19 positivity. Nevertheless, the present study revealed lower infection rates among both the oldest and youngest age groups, which coincided with their high adherence to social distancing measures. These findings are well corroborated by a recent study that found that each 1 unit increase in social distancing was associated with a 26% reduced risk of COVID-19 incidence and a 31% reduced risk of COVID-19 mortality at the county level [13]. Additionally, another study observed that COVID-19 epidemic case growth rates declined by approximately 1% per day, beginning 4 days after the implementation of state-wide social distancing measures [12]. These findings contribute to the existing body of evidence by estimating the impact of social distancing in the community on individual-level outcomes.

### Comparison With Prior Work

Numerous studies have consistently indicated a connection between face mask usage and a decreased risk of COVID-19 at the population level. Specifically, 3 prior studies examining the impact of self-reported face mask use on the risk of COVID-19 reported odds ratios ranging from 0.21 to 0.30, which align with our finding of a 0.36 hazard ratio for always using masks [17-19]. While the protective effect of face masks in reducing transmission to others is well established, our findings suggest that mask use may also reduce the wearer’s exposure to viral load, potentially contributing to decreased infection risk, as previouslyproposed [20].

This study investigated whether demographic variables were associated with a higher COVID-19 positivity rate. However, the logistic regression model did not provide significant evidence of an association between demographic variables (household income, type of residence, education level, race, and number of individuals living in a household) and COVID-19 positivity. The overall model was statistically insignificant, and the predictors included did not substantially contribute to explaining the variability in COVID-19 positivity among participants. Our study results also did not provide evidence of a significant association between living with someone in health care and the COVID-19 positivity rate among participants.

This study also explored whether there is an association between a specific vaccine (Moderna, Pfizer, or Johnson & Johnson) and hospitalization rates. However, we did not find evidence of a significant association between the type of vaccine and hospitalization rates among participants. This indicates that all 3 vaccines are similarly effective in preventing severe outcomes that require hospitalization. It suggests that public health benefits can be achieved with any of the available vaccines and supports their continued use in efforts to reduce the burden of severe illness.

The unprecedented speed and scale of data accumulation on breakthrough infections and related topics have not answered several important questions. For instance, though there is evidence that vaccines against SARS-CoV-2 reduce transmission in households [21] and communities [22], achieving sustained high levels of herd immunity against SARS-CoV-2 infection through vaccination is questioned. This skepticism arises from the mucosal nature of the infection without an obligate stage of dissemination through lymph or blood. Even with high vaccine coverage, a combination of waning immunity and antigenic variation may create enough susceptibility in the population to maintain endemic transmission of SARS-CoV-2, similar to the 4 other coronaviruses circulating in the human population [23]. However, it is unlikely that this situation will result in the same level of disruption seen in the first 2 years of the COVID-19 pandemic. Pandemics are rare events where virtually all humans lack exposure to a novel pathogen, putting them at risk for severe disease and transmission, especially older adult individuals and those with certain comorbidities. Similar to the influenza virus [24], or even more so with human coronaviruses, this pandemic pattern may gradually transition into a pattern of milder disease. Virtually everyone will experience multiple exposures through one or more vaccine doses and/or one or more exposures to viral (possibly breakthrough) infection [25]. In this view, the role of vaccines is not to provide durable herd immunity, as with measles or smallpox, but to prevent severe outcomes during the transition to endemicity.

### Limitations

It is important to note the limitations of the study. This study used convenience sampling, primarily recruiting participants affiliated with academic institutions (eg, students and faculty), which allowed the researchers to focus recruitment on a population that had a known high vaccination rate but may limit the generalizability of the findings. The overrepresentation of younger, female, and White individuals in our cohort (Table 1) could introduce selection bias, as behavioral responses to COVID-19 (eg, mask-wearing and dining out) may systematically differ in older or more diverse populations. Additionally, the lack of diversity in gender, race or ethnicity, age, geographical, and socioeconomic representation among the participants is another limitation that could impact the generalizability of the findings. For instance, our observed high adherence to social distancing among those aged more than 70 years (100%) should be interpreted cautiously, given the small sample size (n=10) in this subgroup. Many participants were affiliated with medical or other health care-related universities, so the participants may have been at higher risk for exposure to COVID-19 infection through occupational or study-related exposures rather than exposures in the community. Participants may have been more knowledgeable about public health guidance and COVID-19 mitigation strategies and more likely to follow masking and social distancing guidelines than the broader population. Since the study was conducted using an anonymous survey, we could not verify responses or the vaccination status of each respondent. Recall bias was mitigated by using validated surveys as a resource and by comparing the behaviors and community settings of respondents who reported testing positive for COVID-19 infection and those who had never tested positive for COVID-19. However, it is possible that there could be some inaccuracies in the responses from both cohorts of respondents. Our analytic approach used separate multivariable models for behavioral and socio-demographic factors rather than a single combined model. This was chosen to reduce collinearity and preserve model stability given the modest sample size. However, this limits our ability to draw conclusions about the independent effects of behavior after adjusting for demographics. Public health measures, such as public masking, social distancing, and reduced maximum occupancy limits for social gathering settings, were variable across the United States, and the degree of adherence to those recommendations was also variable, which could confound the study results regarding which settings were more or less likely to be associated with COVID-19 infection in vaccinated individuals. However, this heterogeneity was distributed evenly among both cohorts of respondents. Future studies should prioritize stratified or population-based sampling to ensure broader applicability of the results.

### Conclusions

In summary, this cross-sectional study aimed to investigate the factors influencing breakthrough COVID-19 infections among vaccinated individuals. The findings revealed significant associations between specific community activities and breakthrough infections in vaccinated individuals, underscoring the importance of targeted interventions. Particularly noteworthy was the consistently higher adherence to social distancing and mask-wearing across various settings among the 18 to 29 years age group, providing valuable insights for the formulation of effective public health strategies. However, the study did not identify significant associations between breakthrough infections and demographic variables, vaccine types, or specific activities related to social distancing and mask-wearing. This underscores the nuanced and multifaceted nature of breakthrough infections, suggesting that individual behaviors and contextual factors play crucial roles. Despite the inherent limitations and challenges in predicting breakthrough infections, this study contributes to our understanding of the complex interplay between vaccination status, community behaviors, and infection risks. The findings highlight the ongoing need for research and flexible public health strategies to adapt to the evolving landscape of the COVID-19 pandemic. As we transition toward endemicity, the role of vaccines becomes pivotal in preventing severe outcomes, and the insights from this study can inform future interventions and policies.

## Supplementary material

10.2196/76679 Multimedia appendices 1 STROBE Checklist

## Abbreviations

CDC: Centers for Disease Control and Prevention
MCPHS: Massachusetts College of Pharmacy and Health Sciences
STROBE: Strengthening the Reporting of Observational Studies in Epidemiology

## References

[1] COVID-19 by county. Centers for Disease Control and Prevention.

[2] Umakanthan S, Sahu P, Ranade AV (2020). Origin, transmission, diagnosis and management of coronavirus disease 2019 (COVID-19). Postgrad Med J.

[3] Durán-Polanco L, Siller M (2021). Crowd management COVID-19. Annu Rev Control.

[4] Wong R, Grullon JR, Lovier MA (2022). COVID-19 risk factors and predictors for handwashing, masking, and social distancing among a national prospective cohort of US older adults. Public Health.

[5] Rahimi F, Talebi Bezmin Abadi A (2020). Criticality of physical/social distancing, handwashing, respiratory hygiene and face-masking during the COVID-19 pandemic and beyond. Int J Clin Pract.

[6] Howard J, Huang A, Li Z (2021). An evidence review of face masks against COVID-19. Proc Natl Acad Sci USA.

[7] Guo Z, Zeng T, Lu Y (2024). Transmission risks of Omicron BA.5 following inactivated COVID-19 vaccines among children and adolescents in China. Commun Med (Lond).

[8] Goodman B (2024). First on CNN: CDC set to stop tracking community levels for Covid-19. CNN.

[9] Panagiotakopoulos L, Moulia DL, Godfrey M (2024). Use of COVID-19 vaccines for persons aged ≥6 months: recommendations of the advisory committee on immunization practices - United States, 2024-2025. MMWR Morb Mortal Wkly Rep.

[10] Buet D, Gallais I, Lauret E (2012). Cotargeting signaling pathways driving survival and cell cycle circumvents resistance to Kit inhibitors in leukemia. Blood.

[11] (2025). Preventing spread of respiratory viruses when you’re sick. Centers for Disease Control and Prevention.

[12] Siedner MJ, Harling G, Reynolds Z (2020). Social distancing to slow the US COVID-19 epidemic: longitudinal pretest–posttest comparison group study. PLoS Med.

[13] VoPham T, Weaver MD, Adamkiewicz G, Hart JE (2021). Social distancing associations with COVID-19 infection and mortality are modified by crowding and socioeconomic status. Int J Environ Res Public Health.

[14] Wrzus C, Hänel M, Wagner J, Neyer FJ (2013). Social network changes and life events across the life span: a meta-analysis. Psychol Bull.

[15] Zhang N, Chen X, Jia W (2021). Evidence for lack of transmission by close contact and surface touch in a restaurant outbreak of COVID-19. J Infect.

[16] Rogawski McQuade ET, Becker L, Stroup SE (2023). Risk factors and titers of COVID-19 infection in a longitudinal statewide seroepidemiology cohort. BMC Infect Dis.

[17] Lalani T, Lee TK, Laing ED (2021). SARS-CoV-2 infections and serologic responses among military personnel deployed on the USNS COMFORT to New York City during the COVID-19 pandemic. Open Forum Infect Dis.

[18] Doung-Ngern P, Suphanchaimat R, Panjangampatthana A (2020). Case-control study of use of personal protective measures and risk for SARS-CoV 2 infection, Thailand. Emerg Infect Dis.

[19] Wang Y, Tian H, Zhang L (2020). Reduction of secondary transmission of SARS-CoV-2 in households by face mask use, disinfection and social distancing: a cohort study in Beijing, China. BMJ Glob Health.

[20] Gandhi M, Beyrer C, Goosby E (2020). Masks do more than protect others during COVID-19: reducing the inoculum of SARS-CoV-2 to protect the wearer. J Gen Intern Med.

[21] Harris RJ, Hall JA, Zaidi A, Andrews NJ, Dunbar JK, Dabrera G (2021). Effect of vaccination on household transmission of SARS-CoV-2 in England. N Engl J Med.

[22] Milman O, Yelin I, Aharony N (2021). Community-level evidence for SARS-CoV-2 vaccine protection of unvaccinated individuals. Nat Med.

[23] Townsend JP, Hassler HB, Wang Z (2021). The durability of immunity against reinfection by SARS-CoV-2: a comparative evolutionary study. Lancet Microbe.

[24] Simonsen L, Clarke MJ, Schonberger LB, Arden NH, Cox NJ, Fukuda K (1998). Pandemic versus epidemic influenza mortality: a pattern of changing age distribution. J Infect Dis.

[25] Lavine JS, Bjornstad ON, Antia R (2021). Immunological characteristics govern the transition of COVID-19 to endemicity. Science.

